# Photocatalyic Appel reaction enabled by copper-based complexes in continuous flow

**DOI:** 10.3762/bjoc.14.251

**Published:** 2018-10-30

**Authors:** Clémentine Minozzi, Jean-Christophe Grenier-Petel, Shawn Parisien-Collette, Shawn K Collins

**Affiliations:** 1 Department of Chemistry and Centre for Green Chemistry and Catalysis, Université de Montréal, CP 6128 Station Downtown, Montréal, Québec, H3C 3J7, Canada

**Keywords:** Appel, continuous flow, copper, halides, photocatalysis

## Abstract

A copper-based photocatalyst, Cu(tmp)(BINAP)BF_4_, was found to be active in a photoredox Appel-type conversion of alcohols to bromides. The catalyst was identified from a screening of 50 complexes and promoted the transformation of primary and secondary alcohols to their corresponding bromides and carboxylic acids to their anhydrides. The protocol was also amendable and optimized under continuous flow conditions.

## Introduction

**Figure 1:**
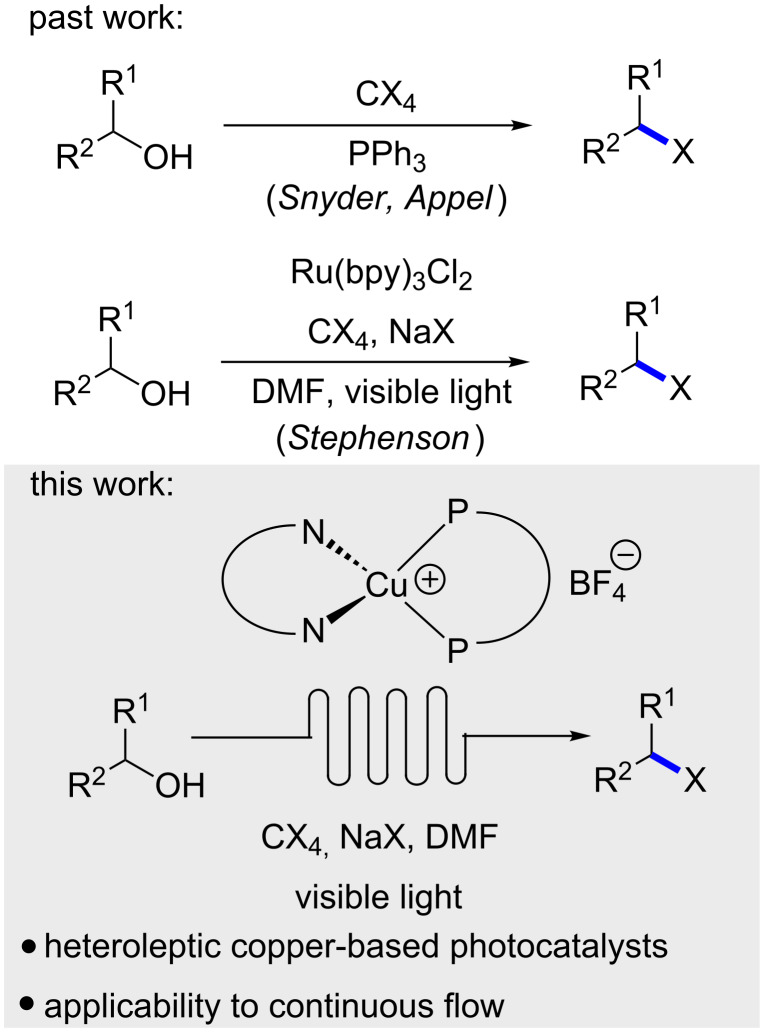
Alcohol→bromide functional group transformations.

Synthetic photochemistry and photocatalysis continues to influence molecular synthesis [1–4]. In exploring photochemical reactivity manifolds, there exists the potential to discover new methods to construct important molecular fragments, as well as revamp traditional chemical transformations. One such process is the Appel reaction [5], which employs PPh_3_ and an electrophilic halogen source to promote the formation of an organic halide from the corresponding alcohol (Figure 1) [6–7]. The Appel reaction is representative of a host of transformations that require stoichiometric reagents to effect a functional group change of an alcohol. In 2011, Stephenson and co-workers reported that photocatalysis could be used to promote the alcohol→halide conversion using low catalyst loadings of a ruthenium-based catalyst (Ru(bpy)_3_Cl_2_, 1 mol %) in the absence of PPh_3_ as a reductant (Figure 1) [8]. The method possesses numerous advantages (wide functional group tolerance, no formation of oxidized phosphine byproducts [9–14], mild reaction conditions and visible-light irradiation), which should be easily embraced by the synthetic community. To further develop the photochemical alcohol→halide transformation, the use of alternative photocatalysts based upon more abundant metals was envisioned [15–18]. Specifically, our group has demonstrated that heteroleptic Cu(I) complexes [19–21] have significant potential as photocatalysts that can promote a variety of mechanistically distinct photochemical transformations including single electron transfer (SET), energy transfer (ET), and proton-coupled electron transfer (PCET) reactions [22–26]. Herein, the evaluation of Cu(I)-complexes for photocatalytic Appel reactions and demonstration in continuous flow is described.

## Results and Discussion

**Figure 2:**
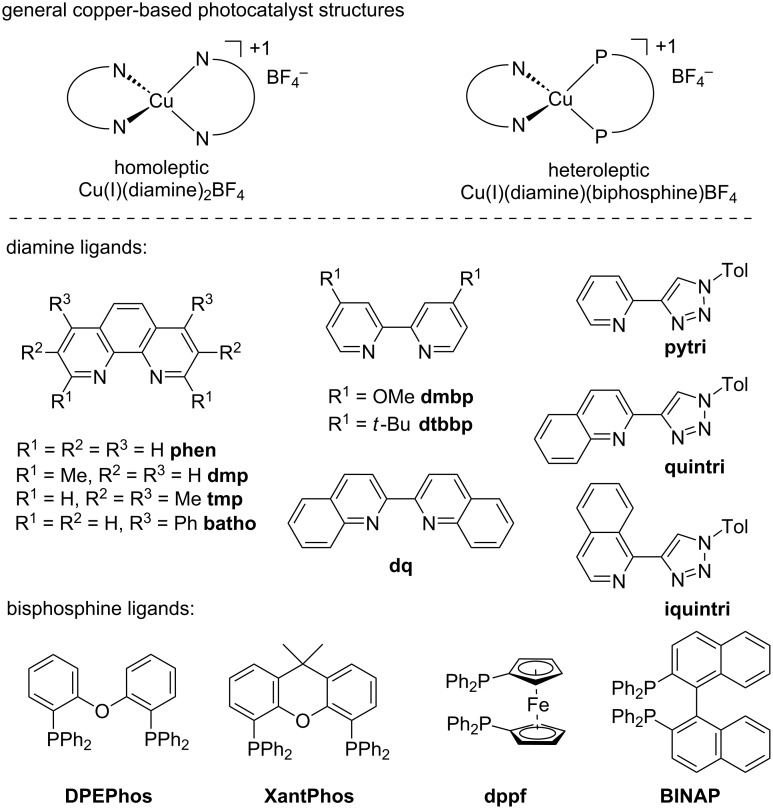
Ligands used in the library generation of heteroleptic copper(I)-based complexes for photocatalysis.

The first step in identifying a heteroleptic diamine/bisphosphine Cu(I)-based photocatalyst for the conversion of an alcohol to bromide involved screening a wide variety of structurally varied complexes. Our group has previously demonstrated that the nature of each ligand influences the physical and photophysical properties as well as catalytic activity of the resulting catalyst (Figure 2) [27].

**Figure 3:**
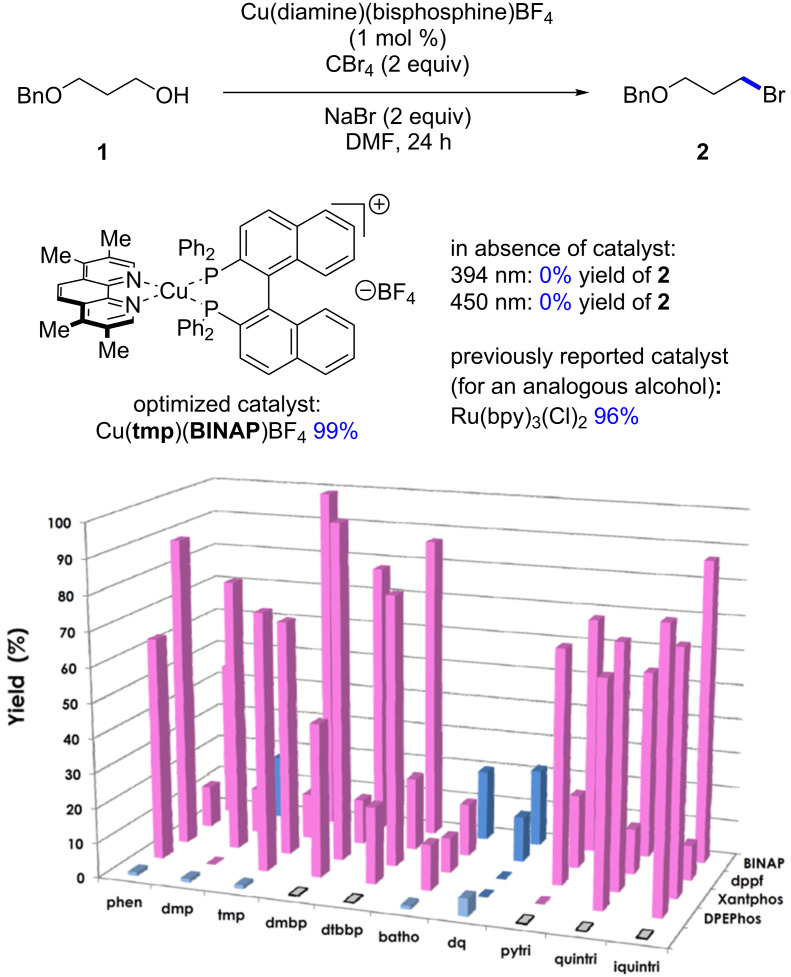
Evaluation of the library of copper-based complexes in photocatalytic alcohol→bromide conversion. Reactions irradiated with 394 nm light (pink) or 450 nm (blue). Front entries without an indicated phosphine ligand pertain to homoleptic Cu(diamine)_2_BF_4_ complexes and are colored in lighter blue. Entries without a color indicate reactions which could not be performed due to solubility or overoxidation of the complex.

A library of 50 different catalysts was evaluated in the conversion of alcohol **1** to bromide **2** (Figure 2 and Figure 3). Several homoleptic complexes were not evaluated due to problematic oxidation or low solubility. Reactions were irradiated at either 394 nm (purple LEDs) or 450 nm (blue LEDs), depending on the UV–vis absorption characteristics of the photocatalysts [28]. Stephenson and co-workers had previously reported that a primary alcohol structurally similar to **1** underwent conversion to the corresponding bromide in 96% yield upon irradiation in the presence of Ru(bpy)_3_Cl_2_ (1 mol %). The screening for a suitable copper-based catalyst was performed under identical reaction conditions whereby the Ru-based photocatalyst was substituted for the Cu-based complex. Control reactions performed in the absence of light or in the absence of catalyst at either 394 or 450 nm revealed no conversion to the bromide. From the results, none of the homoleptic complexes promoted the alcohol-to-halide conversion (**1**→**2**, see the light blue entries in the front row of Figure 3). While many of the heteroleptic complexes promoted the reaction, some trends were apparent. In general, amongst the phosphines the dppf-based complexes were poor catalysts, while when considering the diamine ligands the dq and bathophenthroline catalysts provided poor to modest yields. Also, BINAP and Xantphos-based catalysts tended to afford higher yields of **2**, while amongst the diamines, the triazole-based complexes were almost all efficient at providing **2** (54–87% yield, not including dppf-based complexes). Interestingly, the best catalyst for the transformation (Cu(tmp)(BINAP)BF_4_, 99% of **2**) was a poor catalyst for a previously reported photoredox reaction [27]. It should be noted that Cu(tmp)(BINAP)^+^ possesses an excited state reduction potential of −1.93 V vs. SCE, much greater than that of Ru(bpy)_3_^+2^ (−0.81 V vs SCE), albeit the copper complex has a much shorter excited state lifetime (≈4 ns vs ≈1100 ns for Ru(bpy)_3_^+2^). The excited state reduction potential should match favorably with CBr_4_ (*E*_½_ = 0.30 V vs SCE) in DMF [29]. Note that many of the corresponding homoleptic copper-based sensitizers were ineffective at promoting the Appel-type reaction.

With conditions in hand for the formation of the bromides, different alcohols were converted to their corresponding halides (Table 1). As shown previously, a benzyl-protected alcohol **1** could be transformed to the corresponding bromide **2** in 99% yield, respectively (Table 1, entry 1). The corresponding bromide of citronellol (**4**) was also formed in high yield (91%, Table 1, entry 2). A long chain methyl ester **5** was also tolerated under the reaction conditions (98% of the bromide, Table 1, entry 3). The corresponding dibromide could be formed from 1,9-nonadiol (**6**) in quantitative yield (99%, Table 1, entry 4). A sulfur-containing alcohol **7** was smoothly converted to its bromide in 99% yield (Table 1, entry 5). An allylic alcohol **8** having a *cis*-olefin underwent alcohol-to-halide conversion in 89% yield and was isolated as a 1:1 mixture of *cis* and *trans* isomers (Table 1, entry 6). Finally, a racemic secondary alcohol **9** was easily transformed to the corresponding racemic bromide (99%, Table 1, entry 7).

**Table 1:** Photocatalytic conversion of alcohols to bromides in batch.

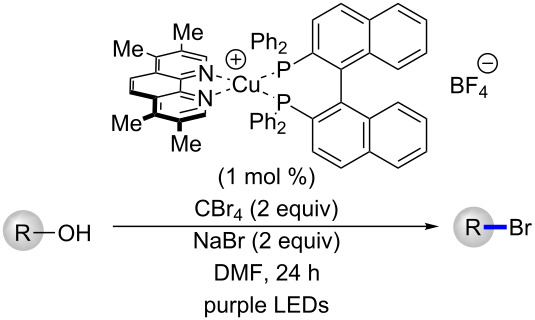			

entry	alcohol		yield (%)^a^

1	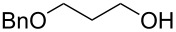	**1**	99
2	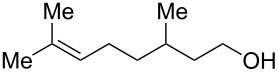	**4**	91
3	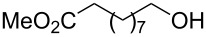	**5**	98
4	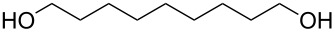	**6**	99
5	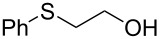	**7**	99
6	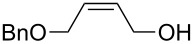	**8**	89^b^
7	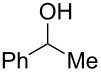	**9**	99

^a^Yield determined by isolation via chromatography. ^b^Isolated as a 1:1 mixture of *cis* and *trans* isomers.

**Figure 4:**
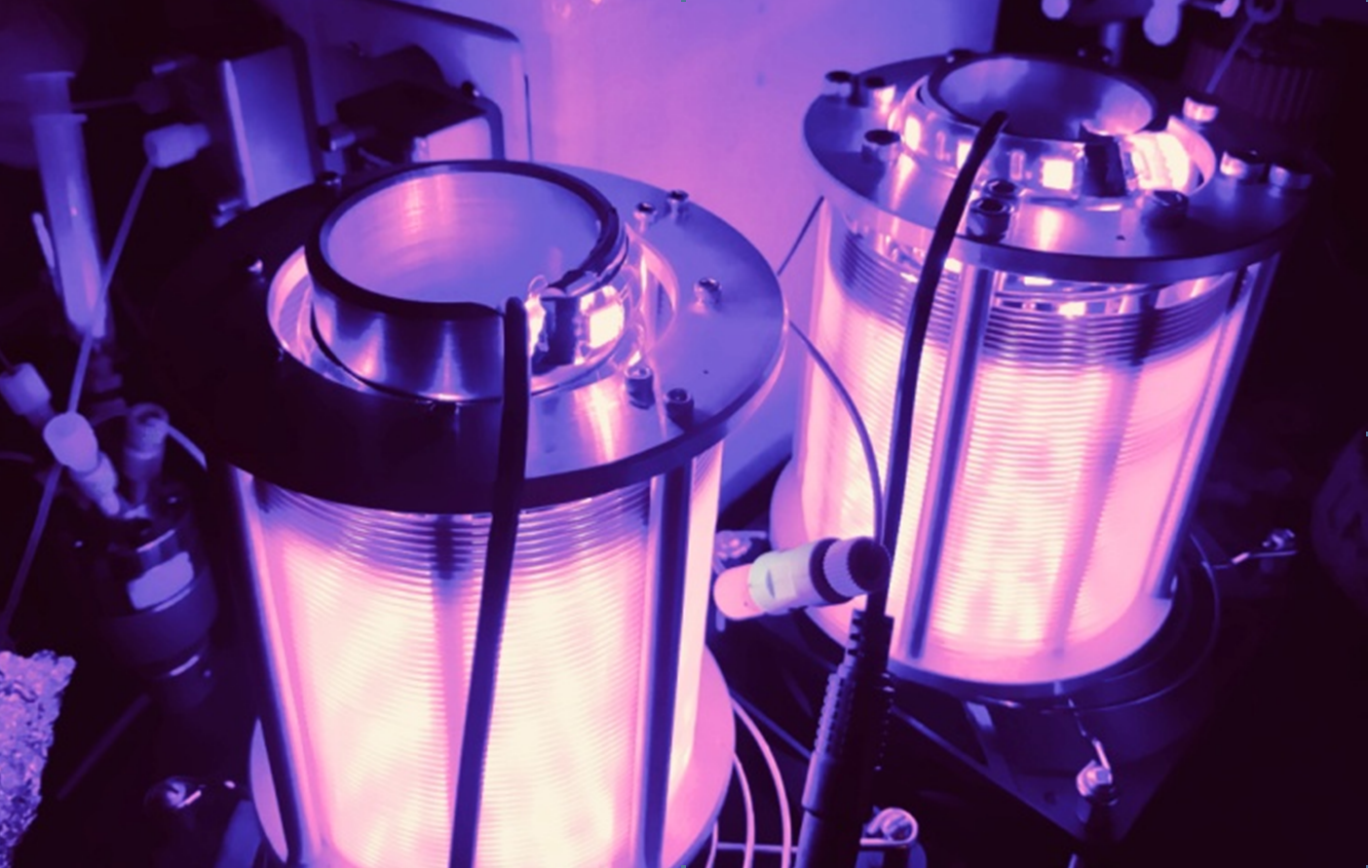
Experimental set-up for the photocatalytic conversion of alcohols to bromides. PFA tubing is wrapped around purple LEDs (394 nm) and fans are placed underneath reactors to maintain cooling.

Following the optimization of the catalyst structure and exploration of scope, the batch reaction conditions were then transferred to continuous flow (Table 2). Initially, an experimental set-up using a previously reported reactor for purple LEDs was selected for the reaction [30–31]. Following injection of the reaction mixture with a target residence time of 60 min, only traces of the desired bromide **2** were observed. Extending the residence time to 120 or 240 min increased the yield to 32–53%, but significant quantities of the starting alcohol **1** and the corresponding formate ester **3** were observed. Using tetra-*n*-butylammonium bromide (TBAB) as the halide source did not improve the yield, but resulted in larger amount of the formylated product **3** (Table 2, entry 4). A possible explanation for the increased yield of **3** when using TBAB could be due to slower displacement of leaving group by the “bulkier” source of bromide. In attempting to extend the residence time, the flow rate of the reaction mixture was decreased. Knowing that faster flow rates can improve mixing and reaction rates [32], an additional reactor was placed in line and the residence time of 240 min was repeated but with an increased flow rate (110 μL/min, Figure 4 and Table 2 entry 5). Gratifyingly, the desired bromide **2** was isolated in 91% yield.

**Table 2:** Photocatalytic conversion of alcohol **1** to bromide **2** in continuous flow.

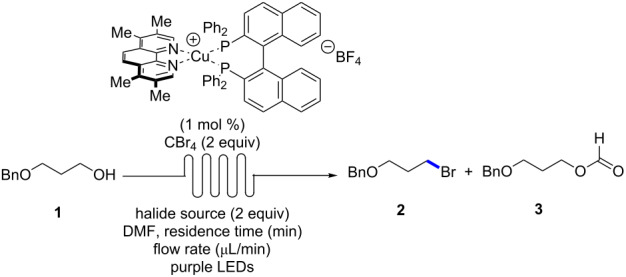						

entry	halide source	*T*_res_ (min)	flow rate (μL/min)	recovered **1** (%)^a^	yield **2** (%)^a^	yield **3** (%)^a^

1	NaBr	60	216	89	<5	<5
2	NaBr	120	110	47	53	–
3	NaBr	240	54.2	45	32	23
4	TBAB	240	110	–	21	63
5	NaBr	240	110	–	91(83)^b^	–

^a^Yield determined by analysis of ^1^H NMR. ^b^Yield determined by isolation via chromatography.

With optimized flow conditions in hand for the formation of bromides in continuous flow, five different alcohols were converted to their corresponding halides (Table 3). The benzyl-protected alcohol **1** could be transformed to the bromide in 83% yield (Table 3, entry 1), as was citronellol (**4**, 83% yield, Table 3, entry 2). A methyl ester **5**, allylic alcohol **8** and racemic secondary alcohol **9** could all undergo conversion to their corresponding bromides in 240 min using the continuous flow protocol (Table 3, entries 3 to 5).

**Table 3:** Photocatalytic conversion of alcohols to bromides in continuous flow.

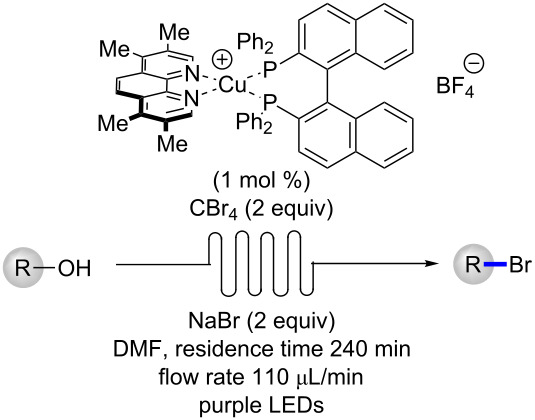			

entry	alcohol		yield (%)^a^

1	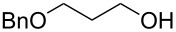	**1**	83
2	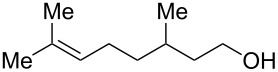	**4**	83
3	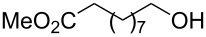	**5**	85
4	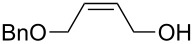	**8**	86
5	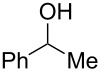	**9**	86

^a^Yield determined by isolation via chromatography.

**Scheme 1:**
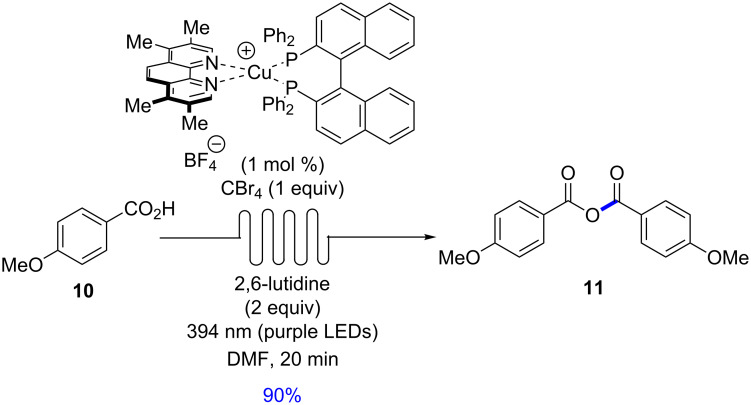
Copper-based photocatalysis for photocatalytic synthesis of an anhydride.

The continuous flow protocol was also applicable to the synthesis of anhydrides, which has also been previously reported by Stephenson and co-workers [33]. The carboxylic acid **10** was submitted to a flow protocol using the optimized Cu(tmp)(BINAP)BF_4_ catalyst, CBr_4_ (1 equiv) and 2,6-lutidine as base with a residence time of 20 min (Scheme 1). The anhydride derived from *p*-methoxybenzoic acid was isolated in 90% yield.

## Conclusion

In summary, a heteroleptic copper-based photocatalyst Cu(tmp)(BINAP)BF_4_ was discovered for the photochemical Appel-type conversion of alcohols to bromides, as well as carboxylic acids to their anhydrides. The protocol was highly efficient and could be adapted to continuous flow using purple LED reactors. The batch and continuous flow processes were all made possible due to the ability to screen highly modular copper-based complexes for photocatalysis.

## Supporting Information

File 1 Experimental details and compound characterization.
