# Supplementary material for: Photocatalyic Appel reaction enabled by copper-based complexes in continuous flow
Source: Beilstein J Org Chem. 2018 Oct 30;14:2730–6. doi: 10.3762/bjoc.14.251 (PMC6244115; doi:10.3762/bjoc.14.251)
Supplement: File 1 — Experimental details and compound characterization. [file Beilstein_J_Org_Chem-14-2730-s001.pdf]

Supporting Information

for

Photocatalyic Appel reaction enabled by copper-based complexes in continuous flow

Clémentine Minozzi, Jean-Christophe Grenier-Petel, Shawn Parisien-Collette and Shawn K. Collins*

Address: Department of Chemistry and Centre for Green Chemistry and Catalysis, Université de Montréal, CP 6128 Station Downtown, Montréal, Québec, H3C 3J7, Canada

Email: Shawn K. Collins - shawn.collins@umontreal.ca

*Corresponding author

Experimental details and compound characterization

TABLE OF CONTENTS:

**GENERAL**	**S2**
**EXPERIMENTAL PROCEDURES AND CHARACTERIZATION DATA**	**S3**

GENERAL :

All reactions that were carried out under anhydrous conditions were performed under an inert argon or nitrogen atmosphere in glassware that had previously been dried overnight at 120 °C or had been flame dried and cooled under a stream of argon or nitrogen. All chemical products were obtained from Sigma-Aldrich Chemical Company or Alfa Aesar and were reagent quality. Technical solvents were obtained from VWR International Co. Anhydrous solvents (CH_2Cl_2, Et_2O, THF, DMF, toluene, and *n*-hexane) were dried and deoxygenated using a GlassContour system (Irvine, CA). Synthesis and characterization of all copper-based catalysts, including the optimized catalyst **Cu(tmp)(BINAP)BF$_4$**, has been previously reported.[1] Isolated yields reflect the mass obtained following flash column silica gel chromatography. Organic compounds were purified using the method reported by W. C. Still[2] and using silica gel obtained from Silicycle Chemical division (40-63 nm; 230-240 mesh). Analytical thin-layer chromatography (TLC) was performed on glass-backed silica gel 60 coated with a fluorescence indicator (Silicycle Chemical division, 0.25 mm, F_{254}.). Visualization of TLC plate was performed by UV (254 nm), $KMnO_4$ or *p*-anisaldehyde stains. All mixed solvent eluents are reported as v/v solutions. Concentration refers to removal of volatiles at low pressure on a rotary evaporator. All reported compounds were homogeneous by thin layer chromatography (TLC) and by ^{1}H NMR. NMR spectra were taken in deuterated $CDCl_3$ using Bruker AV-300 and AV-400 instruments unless otherwise noted. Signals due to the solvent served as the internal standard ($CHCl_3$: δ 7.27 for ^{1}H, δ 77.0 for ^{13}C). The acquisition parameters are shown on all spectra. The ^{1}H NMR chemical shifts and coupling constants were determined assuming first-order behavior. Multiplicity is indicated by one or more of the following: s (singlet), d (doublet), t (triplet), q (quartet), m (multiplet), br (broad); the list of couplings constants (*J*) corresponds to the order of the multiplicity assignment. High-resolution mass spectroscopy (HRMS) was done by the Centre régional de spectrométrie de masse at the Département de Chimie, Université de Montréal from an Agilent LC-MSD TOF system using ESI mode of ionization unless otherwise noted.

[1] Minozzi, C.; Caron, A.; Grenier-Petel, J.-C.; Collins, S. K. *Angew. Chem., Int. Ed.* **2018**, 57, 5477-5481
[2] Still, W. C.; Kahn, M.; Mitra, A. *J. Org. Chem*. **1978**, *43*, 2923.

EXPERIMENTAL PROCEDURES AND CHARACTERIZATION DATA

Procedure for alcohol substrate 1:

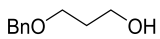

3-(Benzyloxy)propan-1-ol: The Bn-protected alcohol **1** was prepared using a procedure reported by Glimore et al. NMR data was in accordance with what was previously reported.[3]

General procedure for screening of copper catalysts with alcohol 1:

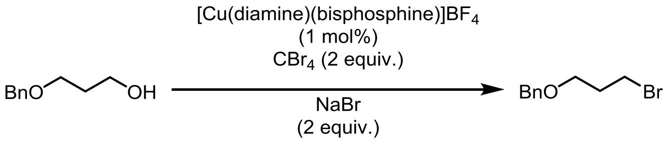

To an open oven-dried reaction vial charged with a stir bar was added copper catalyst (0.002 mmol, 0.01 equiv), the alcohol (33 mg, 0.20 mmol, 1.0 equiv), carbon tetrabromide (67 mg, 0.4 mmol, 2.0 equiv) and sodium bromide (41 mg, 0.40 mmol, 2.0 equiv). The flask vial was capped, purged with a stream of nitrogen and dry DMF (1.5 mL) was added via syringe. The reaction mixture was stirred under LEDs for 24 h. The mixture was poured into a separatory funnel containing Et_2O (10 mL) and H_2O (10 mL). The layers were separated and the aqueous layer was extracted with Et_2O (2 × 10 mL). The combined organic layers were washed with sat. $Na_2S_2O_3$ solution, brine, dried over Na_2SO_4 and concentrated in vacuo. The residue was purified by chromatography (100% Hexanes) to afford the desired product as a colorless oil. NMR data was in accordance with what was previously reported.[4]

---

[3] Plutschack, M. B.; Seeberger, P. H.; Gilmore, K. *Org. Lett.* **2017**, *19*, 30-33.
[4] Dai, C.; Narayanam, J. M. R.; Stephenson, C. R. J. *Nature Chem.* **2011,** *3*, 140-145.

Table S1: Tabular data for the catalytic Appel reaction:

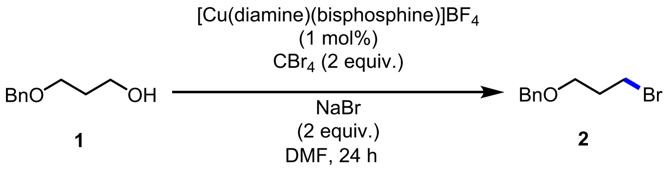

- In absence of catalyst, 380 nm: **0%** yield of **2**
- In absence of catalyst, 450 nm: **0%** yield of **2**

	Yield (%)				
	none	**DPEPhos**	**Xantphos**	**dppf**	**BINAP**
**phen**	1	64	89	12	45
**dmp**	1	0	78	13	18
**tmp**	1	74	68	13	99
**dmbp**	-	44	97	13	78
**dtbbp**	-	22	78	21	87
**batho**	1	13	10	15	20
**dq**	5	0	0	13	22
**pytri**	-	0	67	21	68
**quintri**	-	64	70	13	54
**iquintri**	-	80	70	10	87

Batch reaction:
All photocatalysis batch reactions were performed in 5-dram vials that were placed in the center of an aluminum cylinder in which the interior was lined with a light-emitting diode (LED) strip connected to a power source. LED strips were purchased from Creative Lightings (https://www.creativelightings.com/). No temperature regulation of the reactions was necessary.

Blue LED strips: http://www.creativelightings.com/Sapphire-Blue-LED-Flex-Strips-12vdc-WP-300-Metr-p/cl-frs5050wpdd-5m-12v-bl.htm ;

Purple LED strips: http://www.creativelightings.com/Purple-UV-LED-Flex-Strips-12vdc-WP-300-Meter-p/cl-frs5050wpdd-5m-12v-uv.htm.

General procedure for the Appel reaction in batch:

To an open oven-dried reaction vial charged with a stir bar was added copper catalyst (2 mg, 0.002 mmol, 0.01 equiv), the alcohol (0.20 mmol, 1.0 equiv), carbon tetrabromide (131.6 mg, 0.4 mmol, 2.0 equiv) and sodium bromide (41 mg, 0.40 mmol, 2.0 equiv). The flask vial was capped, purged with a stream of nitrogen and dry DMF (1.5 mL) was added via syringe. The reaction mixture was stirred under purple LEDs (394 nm) for 24 h. The vessel was opened and the mixture was poured into a separatory funnel containing Et_2O (10 mL) and H_2O (10 mL). The layers were separated, and the aqueous layer was extracted with Et_2O (2 × 10 mL). The combined organic layers were washed with sat. $Na_2S_2O_3$ solution, brine, dried over Na_2SO_4 and concentrated in vacuo. The residue was purified by chromatography (100% hexanes).

Procedure for the synthesis of anhydride: To an open oven-dried reaction vial charged with a stir bar was added copper catalyst (2 mg, 0.002 mmol, 0.01 equiv), the *p*-anisic acid (31 mg, 0.20 mmol, 1.0 equiv), carbon tetrabromide (68 mg, 0.2 mmol, 1.0 equiv) and 2,6-lutidine (0.45 µL, 0.40 mmol, 2.0 equiv). The vial was capped, purged with a stream of nitrogen and dry DMF (1.5 mL) was added via syringe. The reaction mixture was stirred under purple LEDs (394 nm) for 12 h. The mixture was poured into a separatory funnel containing Et_2O (10 mL) and H_2O (10 mL). The layers were separated and the aqueous layer was extracted with Et_2O (2 × 10 mL). The combined organic layers were washed with saturated $NaHCO_3$, H_2O, brine sat. $Na_2S_2O_3$ solution, brine, dried over Na_2SO_4 and concentrated in vacuo and concentrated in vacuo to afford the desired anhydride.

For flow reaction:

LED strips were purchased from Creative Lightings (https://www.creativelightings.com/). No temperature regulation of the reactions was necessary.

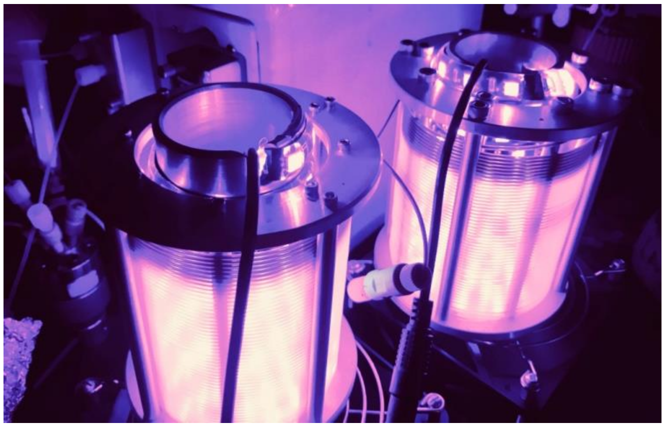

General procedure for the Appel reaction in continuous flow:

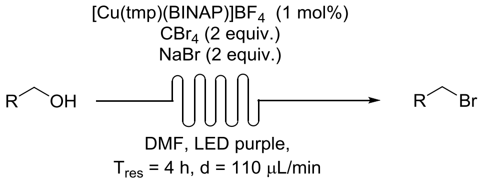

To a 2 mL loop was loaded a solution with copper catalyst (0.002 mmol, 0.01 equiv), the alcohol (0.20 mmol, 1.0 equiv), carbon tetrabromide (131,6 mg, 0.4 mmol, 2.0 equiv) and sodium bromide (41 mg, 0.40 mmol, 2.0 equiv) and 2 mL of dry DMF. The solution was pumped with a Syrris pump through two reactors of PFA tubing would around purple LED strips for a total residence time of four hours and a flow rate 110 µL/min. The eluted reaction mixture was poured into a separatory funnel containing Et_2O (10 mL) and H_2O (10 mL). The layers were separated, and the aqueous layer was extracted with Et_2O (2 × 10 mL). The combined organic layers were washed with sat. $Na_2S_2O_3$ solution, brine, dried over Na_2SO_4 and concentrated in vacuo. The residue was purified by chromatography to afford the desired product.

Characterization of alcohol substrates:

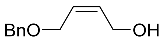

(*Z*)-4-(Benzyloxy)but-2-en-1-ol :NaH (60% in mineral oil, 135.6 g, 5.65 mmol, 1 equiv) was added very slowly to a solution of cis-2-butene-1,4- diol (1 g, 11.3 mmol, 2 equiv) in THF (30 mL) at 0 °C. The resulting mixture was stirred at room temperature for 30 min and then benzyl bromide (0,69 mL, 5.65 mmol, 1equiv) was added. The reaction mixture stirred for 12 h at room temperature. The reaction was quenched with NH_4Cl. The aqueous phase was extracted three times with EtOAc and the combined organic phases were dried over Na_2SO_4. After removal of solvent under reduced pressure the residue was purified by silica gel column chromatography (hexane/AcOEt = 95:5 to 90:10) to give a

yellow oil (313.6 mg, 32%) NMR data was in accordance with what was previously reported.[5]

Characterization of bromides:

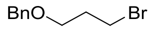

((3-Bromopropoxy)methyl)benzene: Following the general procedure in flow, 3-(benzyloxy)propan-1-ol (33 mg, 0.20 mmol, 1 equiv), carbon tetrabromide (1316 mg, 0.4 mmol, 2.0 equiv) and sodium bromide (41 mg, 0.40 mmol, 2.0 equiv) was dissolved in 2 mL of dry DMF. The crude reaction mixture was purified by chromatography (100% hexanes) to afford the desired product as a yellow oil (38 mg, 83%). NMR data was in accordance with what was previously reported.[6]

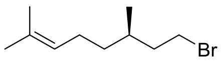

8-Bromo-2,6-dimethyloct-2-ene: Following the general procedure in flow, citronellol (31 mg, 0.20 mmol, 1 equiv.), carbon tetrabromide (131.6 mg, 0.4 mmol, 2.0 equiv) sodium bromide (41 mg, 0.40 mmol, 2.0 equiv) was dissolved in 2 mL of dry DMF. The crude reaction mixture was purified by chromatography (100% hexanes) to afford the desired product as a yellow oil (36 mg, 83%). NMR data was in accordance with what was previously reported.[7]

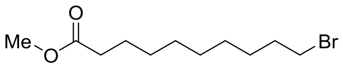

Methyl 10-bromodecanoate: Following the general procedure in flow, methyl 10-hydroxydecanoate (40.5 mg, 0.20 mmol, 1 equiv), carbon tetrabromide (131.6 mg, 0.4 mmol, 2.0 equiv) sodium bromide (41 mg, 0.40 mmol, 2.0 equiv) was dissolved in 2 mL of dry DMF. The crude product was purified by chromatography (100% hexanes) to afford the desired product as a yellow oil (46 mg, 85%). NMR data was in accordance with what was previously reported.[8]

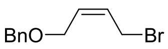

---

[5] Fujiu, M.; Negishi, K.; Guang, J.; Williard, P. G.; Kuroki, S.; Mikami, K. *Dalton Trans*. **2015**, *44*, 19464-19468.
[6] B. C. Schroeder, T. Kurosawa, T. Fu, Y.- C. Chiu, J. Mun, G.- J. N. Wang, X. Gu, L. Shaw, J. W. E. Kneller, T. Kreouzis, M. F. Toney, Z. Bao, *Adv. Funct. Mater.* **2017**, 27, 1701973.
[7] Flachsbarth, B.; Fritzsche, M.; Weldon, P. J.; Schulz, S. *Chem. Biodiv.* **2009,** *6*, 1-37.
[8] Barclay, T. G.; Constantopoulos, K.; Zhang, W.; Fujiki, M.; Matisons, J. G. *Langmuir.* **2012,** *28*, 14172-14179.

(Z)-(((4-Bromobut-2-en-1-yl)oxy)methyl)benzene: Following the general procedure in flow, (*Z*)-4-(benzyloxy)but-2-en-1-ol (35.6 mg, 0.20 mmol, 1 equiv.), carbon tetrabromide (131,6 mg, 0.4 mmol, 2.0 equiv) and sodium bromide (41 mg, 0.40 mmol, 2.0 equiv) was dissolved in 2 mL of dry DMF. The crude reaction mixture was purified by chromatography (100% hexanes) to afford the desired product as a yellow oil (27 mg, 86%). NMR data was in accordance with what was previously reported.[9]

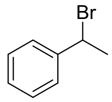

(1-Bromoethyl)benzene: Following the general procedure in flow, 1-phenyl-ethanol (24 μL, 0.20 mmol, 1 equiv.), carbon tetrabromide (131,6 mg, 0.4 mmol, 2.0 equiv) and sodium bromide (41 mg, 0.40 mmol, 2.0 equiv) was dissolved in 2 mL of dry DMF. The crude reaction mixture was purified by chromatography (100% hexanes) to afford the desired product as a yellow oil (27 mg, 86%). NMR data was in accordance with what was previously reported.[10]

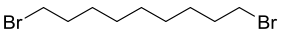

1,9-Dibromononane: Following the general procedure in batch, 1,9-nonanediol (38.4 μL, 0.20 mmol, 1 equiv), carbon tetrabromide (131.6 mg, 0.4 mmol, 2.0 equiv) and sodium bromide (41 mg, 0.40 mmol, 2.0 equiv) was dissolved in 2 mL of dry DMF. The crude reaction mixture was purified by chromatography (100% hexanes) to afford the desired product as a yellow oil (57 mg, 99%). ^{1}H NMR (400 MHz, $CDCl_3$) δ = 3.41 (t, J = 6.9 Hz, 4H), 1.89-1.82 (m, 4H), 1.47-1.40 (m, 4H), 1.34-1.28 (m, 6H); ^{13}C NMR (100 MHz, $CDCl_3$) δ= 34.13, 32.91, 29.36, 28.77, 28.24.

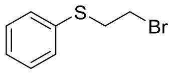

(**2-Bromoethyl)(phenyl)sulfane**: Following the general procedure in batch, 2-(phenylthio)ethan-1-ol (30.8 mg, 0.20 mmol, 1 equiv.), carbon tetrabromide (131.6 mg, 0.4 mmol, 2.0 equiv) and sodium bromide (41 mg, 0.40 mmol, 2.0 equiv) was dissolved in 2 mL of dry DMF. The crude reaction mixture was purified by chromatography (100% Hexanes) to afford the desired product as a yellow oil (43 mg, 99%). NMR data was in accordance with what was previously reported[11]

---

[9] Dai, C.; Narayanam, J. M. R.; Stephenson, C. R. J. *Nature Chem.* **2011,** *3*, 140-145.
[10] Li, Z.; Ebule, R.; Kostyo, J.; Hammond, G. B.; Xu, B. *Chem. Eur. J.* **2017,** *23*, 12739-12743.
[11] Su, T.; Xie, S.; Wei, H.; Yan, J.; Huang, L.; Li, X. *Bioorg. Med. Chem.* **2013**, *21*, 5830-5840.

Synthesis of anhydride 11:

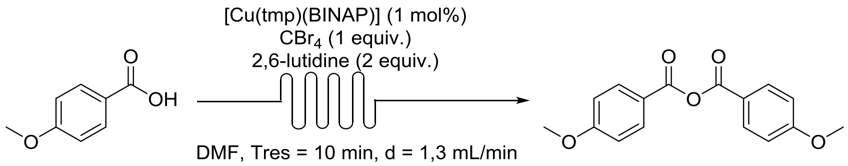

To a 2 mL loop was loaded a solution with copper catalyst (0.002 mmol, 0.01 equiv), the *p*-anisic acid (31 mg, 0.20 mmol, 1.0 equiv), carbon tetrabromide (68 mg, 0.1 mmol, 1.0 equiv) and 2,6-lutidine (0.45 μL, 0.40 mmol, 2.0 equiv) and 2 mL of dry DMF. The solution was pumped through a Syrris pump with a residence time of 20 minutes and a flow rate of 1.3 mL/min. The mixture was poured into a separatory funnel containing Et_2O (10 mL) and H_2O (10 mL). The layers were separated, and the aqueous layer was extracted with Et_2O (2 × 10 mL). The combined organic layers were washed with sat. $Na_2S_2O_3$ solution, brine, dried over Na_2SO_4 and concentrated in vacuo. The anhydride was obtained as a yellow solid (54 mg, 90%). NMR data was in accordance with what was previously reported.[12]

[12] Konieczynska, M. D.; Dai, C.; Stephenson, C. R. J. *Org. Biomol. Chem.* **2012**, *10*, 4509-4511.
